# Clinical Trial Protocol for “Replace Cysto”: Replacing Invasive Cystoscopy with Urine Testing for Non–muscle-invasive Bladder Cancer Surveillance—A Multicenter, Randomized, Phase 2 Healthcare Delivery Trial Comparing Quality of Life During Cancer Surveillance with Xpert Bladder Cancer Monitor or Bladder EpiCheck Urine Testing Versus Frequent Cystoscopy

**DOI:** 10.1016/j.euros.2024.02.018

**Published:** 2024-03-21

**Authors:** Florian R. Schroeck, Robert Grubb, Todd A. MacKenzie, A. Aziz Ould Ismail, Laura Jensen, Gregory J. Tsongalis, Yair Lotan

**Affiliations:** a White River Junction VA Medical Center, White River Junction, VT, USA; b Section of Urology, Dartmouth Hitchcock Medical Center, Lebanon, NH, USA; c Norris Cotton Cancer Center, Dartmouth Hitchcock Medical Center, Lebanon, NH, USA; d The Dartmouth Institute for Health Policy and Clinical Practice, Geisel School of Medicine at Dartmouth College, Lebanon, NH, USA; e Department of Urology, Medical University of South Carolina, Charleston, SC, USA; f Department of Biomedical Data Science, Dartmouth College, Lebanon, NH, USA; g Department of Pathology and Laboratory Medicine, Dartmouth Hitchcock Medical Center, Lebanon, NH, USA; h Department of Urology, University of Texas Southwestern, Dallas, TX, USA

**Keywords:** Bladder cancer, Cancer surveillance, Urinary tumor markers, Bladder–cancer-specific quality of life

## Abstract

“Replace Cysto” is a multisite randomized phase 2 trial including 240 participants with low-grade intermediate-risk non–muscle-invasive bladder cancer, in which participants will be randomized 1:1:1 to one of two urine marker–based approaches alternating a urine marker test (Xpert Bladder Cancer Monitor or Bladder EpiCheck) with cystoscopy or to frequent scheduled cystoscopy. The primary objective is to determine whether urinary quality of life after surveillance is significantly improved in the urine marker arms. The primary outcome will be the patient-reported urinary quality of life domain score of the validated QLQ-NMIBC24 instrument, measured 1–3 d after surveillance. Exploratory outcomes include discomfort after surveillance, the number of invasive procedures that participants undergo per 1000 person years, complications from these procedures per 1000 person years, nonurinary quality of life, acceptability of surveillance, and bladder cancer recurrence and progression. Comparators include surveillance using (1) the Xpert Bladder Cancer Monitor test, (2) the Bladder EpiCheck urinary marker, or (3) frequent cystoscopy alone. After a negative cystoscopy ≤4 mo following bladder tumor resection, all the participants will undergo surveillance at 6, 12, 18, and 24 mo (with time zero defined as the date of the most recent bladder tumor resection). In the urine marker arms, surveillance at 6 and 18 mo will be performed with the marker. Regardless of the arm, participants will undergo cystoscopy at 12 and 24 mo. End of study for each participant will be their 24-mo cystoscopy. Overall trial duration is estimated at 5 yr from when the study opens to enrollment until completion of data analyses. The trial is registered at clinicaltrials.gov (NCT05796375).

## Take Home Message

This trial examines bladder cancer surveillance with non-invasive urine testing versus frequent cystoscopy. Primary outcome is urinary quality of life. If urine testing improves quality of life, we recommend a subsequent phase 3 trial powered for oncologic outcomes.

## 1 Introduction and hypotheses

### 1.1 Current guideline recommendations

As per the current guideline recommendations, patients with non–muscle-invasive bladder cancer (NMIBC) undergo transurethral endoscopic resection followed by surveillance cystoscopy every 3–6 mo [1], making cystoscopy common with over a million procedures per year in the USA [2]. In spite of the burden that frequent cystoscopy procedures place on patients, high-quality data on the benefits of high-intensity surveillance cystoscopy conducted every 3–6 mo are lacking. The 3-mo time interval is due to historical precedent dating back to 1936 [3], and current guideline recommendations are based on expert consensus [1]. The experts writing the American Urological Association guideline recognized the unclear benefit of high-intensity surveillance and specifically stated: “There is an urgent need for studies to determine if less stringent follow-up regimens can be employed without significantly affecting oncological outcomes in these patients” [1].

### 1.2 Urine testing as a less stringent follow-up regimen

One potential strategy to reduce the need for cystoscopy is to replace cystoscopy with less invasive urine-based tumor markers. Preliminary data show that the Xpert Bladder Cancer Monitor test (mRNA based) and Bladder EpiCheck (DNA methylation test) may replace cystoscopy procedures, given their overall sensitivities of, respectively, 0.72 and 0.74 in cross-sectional validation studies [4]. More importantly, their sensitivity to detect high-grade disease is 88% for Xpert Bladder Cancer Monitor and 91% for Bladder EpiCheck. Most urine markers have been used as an adjunct to cystoscopy to help detect “missed” cancer. White light cystoscopy can miss disease in up to 30% due to its overall sensitivity of approximately 70% when compared with enhanced cystoscopy [5]. However, there is potential for urine markers to replace cystoscopy since these have high sensitivity and a high negative predictive value (NPV) for high-grade disease (Table 1). However, patients with a positive or nondiagnostic urine test result will still need to undergo cystoscopy since the urine marker can portend the presence of cancer or the possibility of a false positive finding. With specificity of 0.76 and 0.84 (Table 1) [4], a patient without cancer will have a positive test for up to one in four times. To evaluate the role of urine markers in surveillance of patients, longitudinal comparative data are needed before these tests can be considered for routine care. Specifically, we need evidence that programmatic surveillance with urine markers decreases the burden of surveillance significantly for patients and has acceptable oncological outcomes in diverse populations of bladder cancer survivors.

**Table 1:** Available data on urine tests adapted from recent systematic review [4]

Test	*n* pat.	*n* stud.	Stage a	Grade a	Sensitivity all tumors	Sensitivity high grade	Specificity all tumors	NPV all tumors	NPV high grade
Xpert Monitor	2806	10	Ta: 1274 T1: 452	Low: 1214 High: 818	0.72 (0.63-0.80)	0.88 (0.79–0.96)	0.76 (0.72–0.81)	0.92 (0.90–0.94)	0.99 (0.98–1.00)
EpiCheck	1684	5	Ta: 725 T1: 482	Low: 672 High: 679	0.74 (0.57-0.86)	0.91 (0.85–0.97)	0.84 (0.80–0.88)	0.94 (0.90–0.97)	0.98 (0.96–1.00)

NPV = negative predictive value; pat. = patients; stud. = studies.

Sensitivity and specificity with 95% confidence intervals are shown based on pooled results from meta-analyses [4].

Note that the sensitivity for high grade and the NPV for high grade are based on a corrigendum to the original systematic review [27].

a Not all studies reported stage and grade; thus, the numbers presented here do not always add up to the total number of patients included in the studies [4].

### 1.3 Sparing patients invasive cystoscopy procedures

Replacing cystoscopy with less invasive urine testing will spare bladder cancer patients many of the currently performed cystoscopy procedures. Cystoscopy can be quite uncomfortable for patients [6], as it is performed in nonsedated patients. According to a recent survey of bladder cancer patients, about half of patients experience moderate to severe discomfort during office cystoscopy, and almost 80% consider anxiety a problem associated with the procedure [7]. In a mixed-method study, two-thirds of the participants reported procedural discomfort or worry associated with surveillance cystoscopy [6]. In line with this, the majority of patients undergoing cystoscopy experience the procedure as bothersome and would choose a urine test if proven safe [8].

In preparation of the current protocol, the proposed trial was work-shopped with bladder cancer patients during a breakout session of the Bladder Cancer Advocacy Network’s “Summit” in October 2021. All 22 participants endorsed the study idea as highly or extremely important. This was driven by the negative connotations that cystoscopy brings to patients (word cloud in Supplementary Fig. 1) so that the majority (68%) felt that even avoiding one surveillance cystoscopy through the use of urine testing would be worthwhile.

### 1.4 Observational studies suggest that less intensive cystoscopic surveillance is safe

In our observational study of 1042 patients with low-grade noninvasive Ta bladder cancer from the Department of Veterans Affairs, we found that frequent surveillance was not associated with any improvements in progression or bladder cancer death rates [9]. Similarly, among 1542 patients with high-grade noninvasive Ta bladder cancer from the Department of Veterans Affairs, there was no difference in bladder cancer death when comparing low- versus high-intensity surveillance [10]. This was in line with prior studies demonstrating no survival benefit of high-intensity surveillance using institutional [11] or SEER-Medicare data [12], [13]. These observational data suggest that less stringent follow-up with fewer cystoscopy procedures is probably safe from an oncological perspective. However, changing routine practice to less stringent follow-up by replacing invasive cystoscopy procedures with urine testing will require high-quality longitudinal data from prospective randomized trials such as the one described here.

### 1.5 Urologists and patients are willing to replace cystoscopy with urine testing

One approach to low-intensity surveillance would be to simply decrease the intensity of surveillance cystoscopy, for example, by changing cystoscopy frequency from every 3–6 mo to once a year. However, such an approach could raise a concern among both patients and urologists that recurrent cancer could be missed, because the bladders of these patients would not be checked for an entire year. Replacing cystoscopy with urine testing provides an excellent middle ground between current practice with cystoscopy every 3–6 mo and cystoscopy once a year, as patients’ bladders will still be checked for recurrence while avoiding invasive cystoscopy procedures.

Urologists and patients are willing to replace cystoscopy with urine testing (see Supplementary Table 1) [14], [15]. In the preparation for this study, we discussed with urologists and patients regarding concerns about potentially missing cancer. This led us to choosing a study population of patients with low-grade intermediate-risk (according to the American Urological Association guideline [1]) NMIBC, which has very low rates of cancer progression to more dangerous invasive disease (see section 2.4. for details on these rates). Choosing this population allays concerns about missed cancer and danger associated with avoiding cystoscopy. Furthermore, the high sensitivity of the urinary markers for high-grade disease will result in the detection of nearly all progression or high-grade recurrence events.

## 2 Design

### 2.1 Overview

**Fig. 1:**
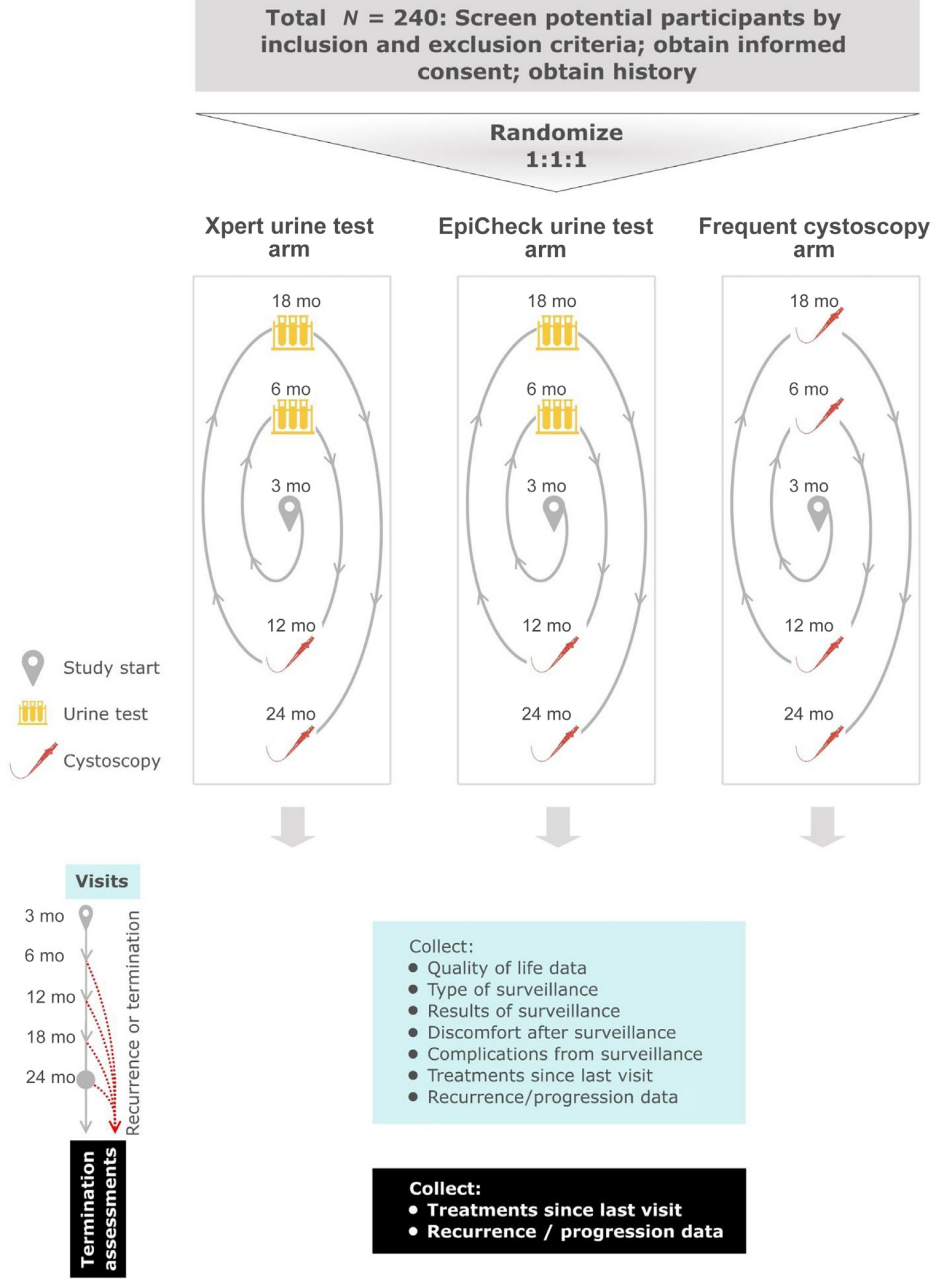
Trial schema.

This is a phase 2 healthcare delivery parallel trial in which participants will be randomized 1:1:1 to programmatic surveillance with the Xpert Bladder Cancer Monitor or the EpiCheck urine test versus frequent cystoscopy at 6, 12, 18, and 24 mo (see the study schema in Fig. 1). Frequent cystoscopy is the standard of care according to current guideline recommendations [1], [16]. In the urine testing arms, urine testing will replace cystoscopy at the 6- and 18-mo marks.

According to the PICOTS principle, this study addresses the following research question: in patients undergoing surveillance for low-grade intermediate-risk NMIBC (population), does programmatic surveillance in which cystoscopy is alternated with either the Xpert Bladder Cancer Monitor or Bladder EpiCheck tests (interventions) compared with frequent cystoscopic surveillance at 6, 12, 18, and 24 mo (comparator) decrease the burden of surveillance (significantly better urinary quality of life; outcomes) when started 3 mo after tumor resection (timing) in diverse outpatient urology practices (setting)?

Our study includes a pragmatic patient-centered design. This was driven by the desire to decrease suffering among bladder cancer survivors by replacing cystoscopy procedures with urine testing. Patients are very interested in this approach, given the burden associated with these procedures [6]. However, if urine tests do not improve quality of life, there is little rationale for further large studies.

We hypothesize that programmatic surveillance with urine testing will lead to significantly better urinary quality of life at the time points of interest at 6 and 18 mo than frequent cystoscopy.

### 2.2 Interventions

Comparators include programmatic surveillance using (1) the Xpert Bladder Cancer Monitor test, (2) the Bladder EpiCheck urine test, or (3) frequent cystoscopy. After a negative cystoscopy ≤4 mo following bladder tumor resection, all the participants will undergo surveillance visits at 6, 12, 18, and 24 mo—a surveillance schedule that is in line with the current guideline recommendations [1], [16]. In the urine testing arms, surveillance visits at 6 and 18 mo will include a visit with the clinician and urine testing with the respective urine test; in case of a positive test result, the participant will undergo an immediate cystoscopy. In the frequent cystoscopy arm, surveillance visits at 6 and 18 mo will include a visit with the clinician and cystoscopy. Regardless of the arm, participants will undergo surveillance visits that include a visit with the clinician and cystoscopy at 12 and 24 mo (see the study schema in Fig. 1). Note that time zero is defined as the date of the most recent bladder tumor resection.

The 12- and 24-mo cystoscopy procedures were included in the urine testing arms for safety reasons. During the planning of this study, both urologists and patients were concerned about “missing cancer.” These cystoscopy procedures will assure that the maximal delay in finding a recurrent cancer in the urine testing arms will be no more than 6 mo. A 6-mo delay is highly unlikely to affect long-term outcomes in this intermediate-risk bladder cancer population, as these patients have only a 1% risk of cancer progression within 1 yr [17].

At the treating clinician’s discretion, any patient is allowed to undergo a for-cause cystoscopy, for example, for gross hematuria or new voiding symptoms. This provides additional reassurance that any participant’s bladder can be inspected directly as per the treating clinician’s discretion if believed to be necessary due to symptoms. Thus, the option to perform for-cause cystoscopy procedures will further minimize the risk of delay in diagnosis of cancer recurrence. Additional bladder cancer clinical evaluations and treatment will also be at the discretion of the treating clinician, and all evaluations and treatments will be recorded.

For the urine tests, participants will provide a voided urine sample during their surveillance visit. Samples will be collected and processed according to manufacturers’ instructions following a defined chain of custody and using standardized laboratory techniques. Currently, the Bladder EpiCheck test is cleared by the US Food and Drug Administration for use in conjunction with cystoscopy [18]. The current trial was approved as a nonsignificant risk device study by the governing institutional review board.

We will run monthly reports for each site to compare whether surveillance was administered according to randomized allocation. For any deviations of >10%, we will approach that site for retraining and technical assistance to optimize adherence to study interventions.

### 2.3 Objective and outcomes

The primary objective is to determine whether urinary quality of life during surveillance is improved in the urine testing arms. We chose the primary outcome, patient-reported urinary quality life, based on (1) importance of the outcome to patients (Table 2) and (2) feasibility and rationale for evaluation in a phase 2 clinical trial. Oncological outcomes (eg, progression of disease) and complications were ranked highest by patients. However, progression and complications are rare and would require a very large study for adequate power. Discomfort was ranked third, and we considered this as the primary outcome. However, we felt that discomfort was too closely linked to the number of cystoscopy procedures that patients undergo in each arm. As such, this would clearly favor the urine testing arms, where patients undergo only two procedures instead of four scheduled procedures over the 2-yr study period (see the study schema in Fig. 1). For these reasons, oncological outcomes, complications, and discomfort were included as exploratory outcomes. Urinary quality of life 1–3 d after surveillance was ranked fourth and was chosen as the most meaningful and feasible primary outcome. For patients with a positive or nondiagnostic urine test, this outcome will be ascertained 1–3 d after the cystoscopy triggered by the urine test.

**Table 2:** Outcomes for the Replace Cysto trial

Rank	Outcome	How included in our study
1	Oncological outcomes, ie, not delaying detection of tumors	Exploratory outcome that will be assessed further in subsequent larger comparative effectiveness study if primary outcome is met
2	Complications of surveillance, eg, infections, strictures	Added as an exploratory outcome, not primary due to rarity of these events
3	Discomfort experienced after surveillance testing	Exploratory outcome, not primary as likely directly related to cystoscopy
4	Burden of surveillance, eg, urinary quality of life, anxiety	Primary outcome: urinary quality of life; exploratory outcome: anxiety
5	Number of cystoscopy procedures	Exploratory outcome

Rank indicates importance assigned by patients and caregivers, with “1” being most important. The five most important outcomes are shown out of a total list of nine reviewed with patients. These are previously unpublished data from workshopping the study with bladder cancer patients during the 2021 Bladder Cancer Advocacy Network’s Bladder Cancer Summit.

### 2.4 Inclusion criteria

Based on discussions with patient advocates and bladder cancer experts, this study will include patients with low-grade intermediate-risk NMIBC, which present approximately a third of all patients. Patients will be enrolled if their surveillance cystoscopy ≤4 mo after bladder tumor resection is negative for recurrent bladder cancer. This population was chosen as it represents the optimal balance between the risk of cancer progression and opportunity to reduce intensity of surveillance.

Regarding cancer progression, urologists and patients may be worried about missing progression in the urine testing arms. In low-grade intermediate-risk patients, this is highly unlikely, as the risk of cancer progression within 6 mo is estimated to be <1% [17]. Furthermore, the time point with the highest risk for recurrence is the 3-mo time point after transurethral bladder tumor resection [19]. Thus, patients will be eligible for the trial only if they have a normal cystoscopy at the 3-mo time point, that is*,* ≤4 mo after the most recent tumor transurethral resection. This will further reduce inclusion of patients with a higher propensity for recurrence and progression of cancer. One additional safety feature is the high sensitivity and NPV of urine markers for patients with high-grade disease, so most progression events would result in a positive test.

Regarding the intensity of surveillance, guidelines recommend cystoscopy procedures every 3–6 mo [1]. Thus, urine testing has the potential to reduce substantially the number of procedures that these patients have to undergo. Currently, intermediate-risk patients undergo at least five surveillance visits in the first 2 yr after tumor resection, providing us adequate longitudinal data to compare the burden of surveillance across arms.

To be eligible to participate in this study, an individual must meet all the following criteria:

1. Aged 18 yr or older.
2. History of low-grade intermediate-risk NMIBC, defined as most recent pathology report showing any of the following:
(a). *Multifocal low-grade* noninvasive urothelial carcinoma of any size
(b). *Solitary low-grade* noninvasive urothelial carcinoma >3 cm in size
(c). *Recurrent low-grade* noninvasive urothelial carcinoma

Note: A history of prior high-grade NMIBC is allowed, as long as the most recent pathology report showing bladder cancer does not show high-grade urothelial carcinoma, carcinoma in situ, or invasive carcinoma.

Note: Papillary urothelial neoplasm of low malignant potential should not be equated to low grade when assessing pathology for inclusion criteria.

3. Stated willingness to comply with all study procedures and availability for the duration of the study
4. No evidence for recurrence at cystoscopy ≤4 mo after the most recent tumor resection
5. Ability to consent in English or Spanish

### 2.5 Exclusion criteria

An individual who meets any of the following criteria will be excluded from participation in this study:

1. History of total cystectomy of the bladder
2. History of urinary diversion (eg, neobladder, colon pouch, or ileal conduit)
3. History of muscle-invasive bladder tumor
4. Known pregnancy or lactation (routine pregnancy testing is not required)
5. History of urothelial carcinoma of the ureter or renal pelvis status after endoscopic treatment or with evidence of recurrent upper tract disease (inclusion allowed for status after nephroureterectomy and recurrence free at the time of inclusion)
6. Anatomic constraints making cystoscopy impossible (eg, history of urethrectomy, obliterated urethra secondary to stricture)
7. Inability to provide a voided urine sample

### 2.6 Schedule of assessments

Participants in the urine testing arms who have a positive or nondiagnostic urine test will undergo cystoscopy no later than 1 mo after the urine test. For these participants, the outcomes listed below will be collected after undergoing a cystoscopy triggered by the positive or nondiagnostic test. Supplementary Table 2 summarizes the schedule of assessments.

#### 2.6.1 Quality of life data (primary and exploratory outcomes) and complications (exploratory outcome)

The primary outcome is the patient-reported urinary quality of life domain score of the validated QLQ-NMIBC24 instrument, measured after surveillance. Exploratory outcomes include other QLQ-C30 and QLQ-NMIBC24 domains as well as anxiety and acceptability. These will be obtained by the clinical research coordinator via a phone survey 1–7 d after consent/randomization and after each surveillance visit via the general QLQ-C30 combined with the bladder cancer–specific QLQ-NMIBC24 [20], via the PROMIS Anxiety Scale [21], and via the Acceptability of Intervention Measure [22]. To encourage participation and to offset the burden of completing the quality of life surveys, participants will receive $20 per completed survey as a small token of appreciation for their time, effort, and inconvenience related to study participation.

For the purpose of collecting quality of life assessments, surveillance visits will be defined by the entire surveillance episode, which will include laboratory processing and communication of test results in the urine testing arms and, if applicable, also a cystoscopy triggered by a positive or nondiagnostic urine test.

#### 2.6.2 Surveillance report

The clinical research coordinator will collect the date of surveillance, type of surveillance performed (ie*,* urine testing or cystoscopy, or standard or enhanced cystoscopy), results of surveillance testing (negative, suspicious, or positive), and next step recommended by the provider. This will allow us to ascertain the type of surveillance that each participant underwent, which will serve as a fidelity measure.

We will also record any evaluations or treatments received since the last visit. This will include dates and results of any for-cause evaluations (eg, cystoscopy or upper tract imaging done for hematuria), and dates and pathology of any bladder biopsies or resections, of any intravesical treatments, and of other bladder cancer–directed therapy. These data will allow us to calculate the number of invasive procedures that each patient underwent during the study period (exploratory outcome).

#### 2.6.3 Surveillance discomfort (exploratory outcome)

The participant will be asked immediately after the cystoscopy or after providing a urine sample (depending on the arm and visit) to rate their discomfort on a visual analog scale ranging from 0 (no pain) to 10 (worst pain).

#### 2.6.4 Bladder cancer recurrence/progression

Bladder cancer recurrence is defined as any bladder abnormality detected during study follow-up, which is biopsied or resected, with local pathology confirming urothelial carcinoma. Note that we require pathological confirmation of recurrence; thus, in-office fulguration without biopsy should not be performed.

Bladder cancer progression is defined as stage or grade progression according to the International Bladder Cancer Group [23], development of metastatic disease, or bladder cancer death. The clinical research coordinator will perform a chart review after each surveillance visit and at participant termination. For participants who developed a recurrence or progression, pathological details of this recurrence or progression will be abstracted from the chart. Following data collection, these participants will be withdrawn from the study and will undergo further treatment and surveillance as per the standard of care.

### 2.7 Study setting and recruitment

Consecutive patients, 18 yr and older, undergoing surveillance for low-grade intermediate-risk bladder cancer will be approached for participation if their first surveillance cystoscopy ≤4 mo after tumor resection shows no evidence for recurrence. We anticipate that the population for this study will include individuals with diverse demographic characteristics from three locations throughout the USA (Charleston, SC; Dallas, TX; and White River Junction, VT). The protocol allows for opening the study at additional sides as needed.

Each site’s principal investigator will present the study protocol to the appropriate local clinical care providers so that those involved in the care of potential participants are familiar with the protocol requirements. Each site will have at least one dedicated clinical research coordinator to facilitate enrollment.

Each site’s principal investigator and clinical research coordinator will work with the clinical care team to identify patients scheduled for surveillance cystoscopy ≤4 mo after tumor resection, who meet the study inclusion criteria. The screening and recruitment procedures will include tracking of all screened and approached patients according to the Screening, Eligibility, Approach, Randomization (SEAR) framework [24].

Electronic health data will be used to identify potentially eligible patients. Specifically, study teams will regularly review lists of patients scheduled for cystoscopy procedures and lists of patients who underwent recent bladder biopsy or transurethral resection. These patients will then be prescreened for potential eligibility by clinical research coordinators using the electronic health record, with data recorded as part of the SEAR framework described above. After preliminary consultation and eligibility assessment by a potential participant’s clinical team, the participating site’s clinical research coordinator will approach each patient immediately after their surveillance cystoscopy to discuss enrollment and informed consent (see the Supplementary material for model informed consent form). We anticipate recruitment of 240 participants over approximately 3 yr.

### 2.8 Randomization

A participant will be enrolled in the study once screened for eligibility and after providing written informed consent. After enrollment, the clinical research coordinator will enter the patient study ID number and stratification factors (site, sex, and receipt of prior intravesical therapy) into the secure online Research Electronic Data Capture (REDCap) system [25] to obtain a study arm assignment. The assignment will be based on the randomization lists provided by the statistician. The list of randomization with 1:1:1 allocation is stratified by site, sex, and receipt of prior intravesical therapy, and was created with permuted blocks of size 6. Receipt of intravesical chemotherapy is defined as treatment with bacillus Calmette-Guérin (BCG) or with intravesical chemotherapy in the 12 mo prior to enrollment.

The clinical research coordinator will then convey the assignment to the treating clinician and participant, and assist with scheduling surveillance visits according to study arm assignment. Given the pragmatic nature of this healthcare delivery study, the participants, clinical staff, and clinical research coordinators cannot be blinded to intervention assignment. However, we will assure that interventions are coded in a way that analytical staff will be blinded to interventions until the analysis phase for the primary outcome is completed.

### 2.9 Data management

Data will be entered into electronic case report forms. The data system includes password protection and internal quality checks, such as automatic range checks, to identify data that appear inconsistent, incomplete, or inaccurate. Clinical data will be entered directly from the source documents. Clinical monitoring will be overseen by an internal oversight committee (IOC). The IOC will include the lead principal investigator, site principal investigator for each of the included sites, study statistician, and central project manager. The IOC will meet via monthly video conference and will review reports for each site to compare whether surveillance was administered according to randomized allocation.

The central project manager will conduct random monitoring of target data, including, but not limited to, verification of signed consent documents, inclusion criteria, phone survey data, type of surveillance, and results of surveillance. The IOC will oversee central monitoring. This will include assessing the following for each site: data entry errors, missing data, and responsiveness to data queries from the central study team. In addition, the IOC will assign the cause of death for any patients who die during study follow-up. Site investigators and clinical research coordinators will be contacted as needed for retraining and technical assistance if systematic data integrity issues are discovered at their site. The IOC will review and approve a monthly data integrity report, which will be stored for record keeping.

## 3 Statistics

### 3.1 Power calculations

The hypothesis tests of primary interest are whether urinary quality of life scores are improved 1–3 d after surveillance with urine testing relative to surveillance with frequent cystoscopy. Statistical testing will be performed to show that at least one of the urine test arms is superior to the control arm at a Bonferroni corrected 0.025 type 1 error rate.

We calculated power using a simulation coded in R (Table 3). The simulations were conducted with the following settings. The study consists of three equally allocated arms with five time points. The time points of primary interest are the second (6 mo) and fourth (18 mo) when urine tests are done in the Xpert Bladder Cancer Monitor and Bladder EpiCheck arms, whereas cystoscopy is done in the frequent cystoscopy arm. The dependent variable is the QLQ-NMIBC24 urinary domain score and assumed to be normally distributed, conditional on the structural part of the model. The correlation structure of the five time points is assumed to be exchangeable. The correlation (ie, intraclass correlation coefficient) of this exchangeable matrix is assumed to be 0.7 based on the available data for the urinary domain score of the QLQ-NMIBC24 [26]. The structural model assumed is that the dependent variable will be on average β units higher (the effect size) following a urine test than after a cystoscopy. For participants with a positive or nondiagnostic urine test at 6 or 18 mo, a cystoscopy will be performed within 1 mo for further evaluation. For these participants, the dependent variable for the 6- or 18-mo time point is the urinary domain score after the cystoscopy. The simulations assume the Xpert Bladder Cancer Monitor test has sensitivity of 72% and specificity of 76%, whereas the EpiCheck has sensitivity of 74% and specificity of 84% [4], [27].

**Table 3:** Minimum detectable effect size for improvement in urinary quality of life domain score for superiority of at least one of the two urine test arms

SD = standard deviation.

Note that values were rounded to the nearest 0.05, which creates apparent equivalences in some cells.

We assumed 20% missing data for the primary outcome at each time point. In line with prior work [28], we assume that cancer recurs at a rate of 10%/yr (exponential distribution). Patients see bladder cancer surveillance visits as the highest priority [29]—as such attrition is expected to be low. However, we have conservatively assumed a 10% attrition rate per year.

The simulation is based on the power to show that at least one of the urine test arms is superior to the frequent cystoscopy arm at a Bonferroni corrected 0.025 type 1 error rate, within a mixed-effect model with a random effect for the participant and a structural model with a main effect for the 6-, 12-, 18-, and 24-mo time points and their interaction with the study arm. This is similar to the power of a multivariate Wald test of the equivalence of all three arms at a type 1 error rate of 5%.

Under these assumptions, our study of 240 participants (80 participants per arm) has sufficient power (80%) to detect an effect size of 0.6 standard deviations (green cell in Table 3). This corresponds to a concordance (Mann-Whitney proportion) of 66%. That is, we assume that 66% of the time, a participant who was randomized to a urine testing arm will have a significantly better urinary domain score at 6 or 18 mo than a person who was randomized to the frequent cystoscopy arm.

We acknowledge that the trial is not adequately powered to address some of the exploratory outcomes, for example, complications, bladder cancer recurrence, and bladder cancer progression, given the rarity of these outcomes. Analyses will account for sex as a biological variable using it as a potential covariate in statistical models and subgroup analyses. Given that this is a phase 2 healthcare delivery trial, we will have limited power to determine whether sex modifies the treatment effect. However, if women had zero change in urinary quality of life with urine testing while men would have an improvement of 1.4 standard deviations on the urinary symptom domain scale, we would have 80% power to detect the heterogeneity of treatment effect.

### 3.2 Analyses plans

#### 3.2.1 Populations for analyses

The intent-to-treat population is defined as the population of participants who were randomized to any of the study arms. The participants will be categorized (in terms of their intervention assignment) based on their initial randomized group and will be included in analyses irrespective of their status—completer or dropout before completion.

The safety population includes all the participants who were consented to the trial, randomized to any of the arms, and completed at least one postrandomization surveillance visit.

#### 3.2.2 Primary outcome analyses: urinary quality of life

The primary outcome is the patient-reported urinary quality of life domain score of the validated QLQ-NMIBC24 instrument [20], measured as a repeated measure at each surveillance visit. We will model the primary outcome urinary quality of life in terms of the study arm and time using a mixed-effect model with a random intercept for the participant. We will include fixed effects for the study arm (indicators for the Xpert Bladder Cancer Monitor and the EpiCheck urine test with the frequent cystoscopy arm as the referent, ie*,* intent–to-treat analyses), for time points (3, 6, 12, 18, and 24 mo) as categorical variables (3-mo baseline as referent), and the interactions of the study arm and time point. The interaction provides information about the treatment efficacy and is analogous to a difference in difference. We will also add a fixed effect for sex as a biological variable, given that women’s urethras are much shorter than men’s, which may very well affect urinary quality of life after surveillance testing. Results from these models will be presented as adjusted means with standard deviations.

As a complementary analysis, we will employ a generalized estimating equation (GEE) with the same fixed effects and cluster with respect to the participant using an autoregressive 1 correlation structure. As another evaluation of the robustness of findings, we will repeat the mixed effects and GEE analysis above, removing the 3-mo baseline value from the longitudinal vector of urinary quality of life and instead add it as a covariate (fixed effect) and eliminate the interaction from the model; the information on efficacy from these complementary analyses is in the main effects for the study arm. If these complementary analyses do not confirm the findings from the primary analyses, we will explore the reasons for the divergent results and report these with our main study findings.

The hypothesis tests of primary interest are whether urinary quality of life scores are improved 1–3 d after surveillance with urine testing relative to surveillance with frequent cystoscopy. For participants with a positive or nondiagnostic urine test, this outcome will be ascertained 1–3 d after the cystoscopy triggered by the urine test. Each of the two urine test modalities will be compared with the frequent cystoscopy arm, using the Bonferroni-Holm method [30] to control the family-wise type 1 error rate at 5%. Each of these two tests (each urine test vs frequent cystoscopy arm) will be conducted using a multivariate Wald test that averages the corresponding coefficients from the 6- and 18-mo time points from the interaction of the study arm with time points. If there is a significant effect of urine test modalities (done at 6 and 18 mo) compared with the frequent cystoscopy arm, we will test whether the effect is different at 6 versus 18 mo.

All analyses will be performed using intent-to-treat principles and within the intent-to-treat population. For example, if a patient in a urine test arm does not complete a urine test and has a cystoscopy instead at 6 or 18 mo, we will nonetheless analyze this patient’s outcome data from this time point as in a urine test arm.

We do not anticipate that missing data will be a major issue as each site will have at least one staff member dedicated to obtaining the primary outcome data, that is, validated urinary quality of life domain scores, 1–3 d after the surveillance visit. Based on a similar prior longitudinal study validating the QLQ-NMIBC24 [20], we assume 20% missing data for the primary outcome at each time point.

Missing outcomes can be analyzed as is using the mixed-effect model for longitudinal data described above, under the assumption that the distribution of the outcome in participants for whom it is not missing does not differ from the distribution in those in whom it is missing conditional on previous observations of the outcome and covariates. We will also carry out sensitivity analyses to determine to what extent conclusions are sensitive to departures from this missing at random assumption. Our longitudinal analytic approach described above allows us to incorporate study data even if study visits were missed or participants were lost to follow-up.

#### 3.2.3 Subgroup analyses

We will add a fixed effect for sex as a biological variable into our analytic models, given that women’s urethras are much shorter than men’s, which may very well affect urinary quality of life after surveillance testing. Results from these models will be presented as adjusted means with standard deviations, stratified by sex.

Similarly, we will add a fixed effect for race into our analytic models and present results as adjusted means with standard deviations, stratified by race.

Additional subgroup analyses may be added to the statistical analysis plan. These may also be performed post hoc if approved by the IOC.

Given that this is a phase 2 healthcare delivery trial, we will have limited power to determine whether sex modifies the treatment effect. Similarly, power will be limited for any other subgroup analyses.

#### 3.2.4 Exploratory outcome analyses

For the continuous patient-reported outcome scales, we will use linear mixed-effect models as described for the primary outcome.

We will evaluate complications after surveillance procedures and the number of invasive procedures as a count, that is, the number of complications per time under surveillance or the number of invasive procedures per time under surveillance. For these outcomes, we will use mixed effects and Poisson regression with a random intercept for each participant and the same fixed-effect model structure as described for the primary outcome.

For recurrence and progression, which are time to event, we will plot cumulative incidence curves, because this endpoint is subject to censoring by attrition. We will report cumulative incidence for each arm at 12 and 24 mo and compare incidence using a three-sample log-rank test. For consistency with the existing literature [17], time zero will be the time of each patient’s last tumor resection, and the time for any event will be the time of pathologically confirmed recurrence or progression. We will use the Fine-and-Gray approach to calculate proportional subdistribution hazards to address any competing risk of death [31].

**Fig. 2:**
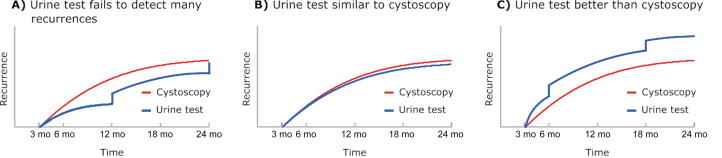
Possible patterns of recurrence that will be evaluated in exploratory analyses. (A) If the urine test fails to detect many recurrences, there will be a substantial step-up at the 12- and 24-mo cystoscopy time points. (B) If the urine test is similar to cystoscopy, the curves will have similar shapes. (C) If the urine test is better than cystoscopy, there will be a substantial step-up at the 6- and 18-mo urine testing time points.

Furthermore, we will estimate important feasibility data for a subsequent large noninferiority comparative effectiveness trial. We will use data from the data management and reporting system to estimate screening to enrollment ratios as well as dropout and crossover rates. Finally, we will plot patterns of recurrence and progression over time for the urine testing arms versus control, which will provide important preliminary data for powering the subsequent large noninferiority comparative effectiveness trial (Fig. 2). These longitudinal patterns are important to understand, as reporting of positive urine test results may make subsequent cystoscopy more sensitive because providers perform a more thorough evaluation after a positive test (Fig. 2C) [32]. This could signal an advantage of urine testing over control, but could lead to higher estimated recurrence rates in the urine testing arms, which would be hard to interpret without longitudinal data.

### 3.3 Reporting of adverse events

Adverse events (AEs) will be tabulated for the safety population as proportions with binomial 95% confidence intervals according to the type of surveillance performed as follows: The denominator will be the number of surveillance visits using the Xpert Bladder Cancer Monitor test, the EpiCheck urine test, or cystoscopy. The numerator will be the number of surveillance visits that resulted in a specific AE (eg, gross hematuria, urinary tract infection, etc.) for each type of surveillance performed. Each specific AE will be counted only once per visit. We will report these proportions without formal significance testing, because we anticipate that AEs will be rare and to avoid type 1 errors due to multiple testing.

### 3.4 Stopping rules for evidence of harm

We will follow stopping rules if there is evidence that urine testing increases recurrence or progression.

For recurrence, we will calculate the cumulative incidence of recurrence for each arm when half of the total follow-up has accumulated. We anticipate that this analysis will happen approximately 1 yr after half of the participants have been enrolled. We will compare the cumulative incidence in the urine testing arms with that in the frequent cystoscopy arm using a log-rank test. If the cumulative incidence of recurrence is statistically significantly higher in one of the urine testing arms than in the frequent cystoscopy arm with an alpha level of 0.05, then the respective urine testing arm will be closed for enrollment and all the participants from that arm will be transitioned to the standard of care. Based on power calculations, this would require a hazard ratio of ≥3.2.

We will assume that progression to more dangerous disease was missed at an unacceptable rate by urine testing if four participants in a urine testing arm develop progression to muscle-invasive disease that is detected at the 12- or 24-mo cystoscopy visit, but was not detected with the preceding 6- or 18-mo respective urine tests. If four participants in the Xpert Bladder Cancer Monitor test arm fit this scenario, then the Xpert urine test arm will be closed for enrollment and all the participants from that arm will be transitioned to the standard of care. If four participants in the EpiCheck urine test arm fit this scenario, then the EpiCheck urine test arm will be closed for enrollment and all the participants from that arm will be transitioned to the standard of care.

## 4 Summary

To our knowledge, this is the first prospective trial conducted in the USA to provide level 1 evidence on standard versus less intensive surveillance of NMIBC using novel urine tests. Thus, our trial is poised to disrupt a paradigm of high intensity surveillance that has existed for >80 yr without high-quality evidence. According to the current (2016, amended 2020) national guidelines [1], high-intensity surveillance is supported by “panel consensus and historic precedence” only. High-intensity surveillance every 3–6 mo has been recommended since 1936, based on the believe that it takes the bladder ∼3 mo to heal after transurethral resection of a bladder tumor [3]. Our “Replace Cysto” trial will evaluate whether NMIBC patients’ quality of life can be improved significantly in the surveillance phase by reducing the intensity of surveillance with novel urine tests.

Our study is innovative because it will be the first longitudinal head-to-head comparison of two novel molecular urine tests and because it is focused on outcomes that matter to patients. The studies providing data on test characteristics (Table 1) have largely been funded by the manufacturers of these tests. Our Replace Cysto trial will provide an independent National Institutes of Health–funded evaluation of these tests and will focus on outcomes that matter to patients.

Importantly, our study is focused on the National Cancer Institute’s scientific priority area of cancer survivorship. The number of cancer survivors in the USA is projected to grow dramatically over the next decades, from 15.5 million in 2016 to 26.1 million in 2040 [33], and bladder cancer is the fifth most prevalent cancer overall [34]. We will systematically study whether the burden of surveillance cystoscopy can be improved among bladder cancer survivors by replacing invasive cystoscopy procedures with urine testing. These procedures are associated with substantial anxiety [6]. In our recent survey of 392 bladder cancer survivors, cystoscopy was associated with substantial discomfort in 52%, and almost 80% considered anxiety a problem associated with the procedure [7]. Less intensive surveillance with urine testing would spare survivors up to half of all invasive cystoscopy procedures, potentially substantially reducing their burden of surveillance.

We purposefully kept the eligibility criteria broad so that the results of our trial are applicable to a bladder cancer surveillance population. For example, patients who have a history of hematuria or urinary tract infections will not be excluded. For the EpiCheck urine test, data submitted to the Food and Drug Administration show that there was no interference of 27 potentially interfering substances including common urine constituents and microbial contaminants [18]. The Xpert Bladder Cancer Monitor test has been validated among patients with hematuria who present for diagnostic workup [35]. Thus, it is unlikely that hematuria would negatively affect its performance. We would also like to note that in keeping with the pragmatic nature of the trial, cytology is neither mandated nor excluded. If clinicians desire to obtain cytology, this is allowed. If cytology is abnormal and prompts a for-cause cystoscopy, this information will be collected as the cause of the for-cause cystoscopy. However, cytology has low sensitivity (∼12–16%) for low-grade disease [36], [37]. Given that the included population will be intermediate-risk low-grade NMIBC patients, mandating routine cytology would be of low yield.

It is worth mentioning that the “Urofollow” trial was conducted recently in Europe. There are two main differences between Urofollow and our trial: (1) the Urofollow trial has only two arms: a standard arm and a urine testing arm; and (2) the Urofollow trial employs a battery of urine tests in the urine testing arm, including UroVysion, uCyt+, NMP22, and urine cytology. In addition, participants in the urine testing arm will also undergo ultrasound examination of the bladder [38]. Urofollow also explores a number of newer urine tests “ex posteriorim,” including the Xpert Bladder Cancer Monitor test [38]. Conversely, our Replace Cysto trial has two distinct urine testing arms and is focused on comparing quality of life outcomes between surveillance with frequent cystoscopy versus with two commercially available tests (the Xpert Bladder Cancer Monitor and EpiCheck urine tests).

In summary, our patient-centered approach has the potential to decrease suffering among bladder cancer survivors by replacing cystoscopy procedures with urine testing. Patients are very interested in this approach given the burden associated with these procedures. However, if urine tests do not improve quality of life, there is little rationale for further large studies. If they significantly improve the quality of life, our data will provide a strong justification for a subsequent comparative effectiveness phase 3 trial of programmatic surveillance with urine testing versus standard cystoscopy that is large enough to be powered for oncological outcomes.

  ***Author contributions*:** Florian R. Schroeck had full access to all the data in the study and takes responsibility for the integrity of the data and the accuracy of the data analysis.

  *Study concept and design*: Schroeck, Lotan.

*Acquisition of data*: None.

*Analysis and interpretation of data*: None.

*Drafting of the manuscript*: Schroeck.

*Critical revision of the manuscript for important intellectual content*: Schroeck, Grubb, MacKenzie, Ould Ismail, Jensen, Tsongalis, Lotan.

*Statistical analysis*: None.

*Obtaining funding*: Schroeck.

*Administrative, technical, or material support*: Schroeck, Ould Ismail, Jensen.

*Supervision*: Schroeck.

*Other*: None.

  ***Financial disclosures:*** Florian R. Schroeck certifies that all conflicts of interest, including specific financial interests and relationships and affiliations relevant to the subject matter or materials discussed in the manuscript (eg, employment/affiliation, grants or funding, consultancies, honoraria, stock ownership or options, expert testimony, royalties, or patents filed, received, or pending), are the following: Yair Lotan is a consultant for Nanorobotics, C2I Genomics, Photocure, AstraZeneca, Merck, Fergene, Abbvie, Nucleix, Ambu, Seattle Genetics, Hitachi, Ferring Research, Verity Pharmaceuticals, Virtuoso Surgical, Stimit, Urogen, Vessi Medical, CAPs Medical, Xcures, BMS, Nonagen, Aura Biosciences, Inc., Convergent Genomics, Pacific Edge, Pfizer, Phinomics Inc, CG Oncology, Uroviu, On Target Lab, Promis Diagnostics, Valar Labs, and Uroessentials. Florian R. Schroeck receives research funding from Pacific Edge, Ltd.

  ***Funding/Support and role of the sponsor*:** This trial is supported by the National Institutes of Health, National Cancer Institute (grant R37CA275916), and by Nucleix Inc., which provides urine testing for participants free of charge, as well as by Cepheid, who supports urine testing for participants via research funds and materials. The funding organizations had no role in the design and conduct of the study; and will have no role in collection, management, analysis, and interpretation of the data. They had no role in the preparation of the manuscript and in the decision to submit the manuscript for publication. Nucleix and Cepheid were provided versions of the manuscript for comment prior to publication, as specified in the respective Cooperative Research and Development Agreements. Opinions expressed in this manuscript are those of the authors and do not constitute official positions of the US Federal Government, the Department of Veterans Affairs, the Institutes of Health, or the National Cancer Institute.

  ***Acknowledgments*:** This study was supported using resources and facilities at the White River Junction Department of Veterans Affairs (VA) Health Care System. Xpert Bladder Cancer Monitor is a urinary biomarker test intended to monitor for the recurrence of bladder cancer in patients previously diagnosed with bladder cancer (CE-IVD In Vitro Diagnostic Medical Device). It may not be available in all countries and is not available in the USA.

  ***Ethics statement*:** This study is approved by the WCG IRB (Study #1352809). For the protocol presented here, no data on individual participants are presented; thus, consent to participate did not apply.

  ***Data sharing statement*:** All data generated or analyzed during this study are included in this published article and its supplementary information files. Once the trial is complete and analyzed, trial data will be provided via a National Cancer Institute–approved data repository.

## References

[1] Chang SS, Boorjian SA, Chou R, et al. Non-muscle invasive bladder cancer: American Urological Association/SUO guideline. 2016. https://www.auanet.org/education/guidelines/non-muscle-invasive-bladder-cancer.cfm.

[2] The Dartmouth Institute for Health Policy & Clinical Practice. Dartmouth atlas of health care - coding trends. 2018. https://atlasdata.dartmouth.edu/static/supp_research_data#coding_trends.

[3] Levy DA, Jones JS. History of cystoscopy. In: Lokeshwar VB, Merseburger AS, Hautmann SH, editors. Bladder tumors: molecular aspects and clinical management. Chapter 10.3. Springer Science & Business Media; 2010. p. 194.

[4] Laukhtina E., Shim S.R., Mori K. (2021). Diagnostic accuracy of novel urinary biomarker tests in non-muscle-invasive bladder cancer: a systematic review and network meta-analysis. Eur Urol Oncol.

[5] Jocham D., Stepp H., Waidelich R. (2008). Photodynamic diagnosis in urology: state-of-the-art. Eur Urol.

[6] Koo K., Zubkoff L., Sirovich B.E. (2017). The burden of cystoscopic bladder cancer surveillance: anxiety, discomfort, and patient preferences for decision making. Urology.

[7] Kukreja J.B., Schroeck F.R., Lotan Y. (2022). Discomfort and relieving factors among patients with bladder cancer undergoing office-based cystoscopy. Urol Oncol.

[8] Vriesema J.L.J., Poucki M.H., Kiemeney L., Witjes J.A. (2000). Patient opinion of urinary tests versus flexible urethrocystoscopy in follow-up examination for superficial bladder cancer: a utility analysis. Urology.

[9] Schroeck F.R., Lynch K.E., Li Z. (2019). The impact of frequent cystoscopy on surgical care and cancer outcomes among patients with low-risk, non-muscle-invasive bladder cancer. Cancer.

[10] Rezaee M.E., Lynch K.E., Li Z. (2020). The impact of low- versus high-intensity surveillance cystoscopy on surgical care and cancer outcomes in patients with high-risk non-muscle-invasive bladder cancer (NMIBC). PLoS One.

[11] Lee C.T., Dunn R.L., Ingold C., Montie J.E., Wood D.P. (2007). Early-stage bladder cancer surveillance does not improve survival if high-risk patients are permitted to progress to muscle invasion. Urology.

[12] Hollenbeck B.K., Ye Z., Dunn R.L., Montie J.E., Birkmeyer J.D. (2009). Provider treatment intensity and outcomes for patients with early-stage bladder cancer. J Natl Cancer Inst.

[13] Hollingsworth J.M., Zhang Y.S., Miller D.C. (2011). Identifying better practices for early-stage bladder cancer. Med Care.

[14] Sayyid R.K., Sayyid A.K., Klaassen Z. (2018). Replacing surveillance cystoscopy with urinary biomarkers in followup of patients with non-muscle-invasive bladder cancer: patients’ and urologic oncologists’ perspectives. Can Urol Assoc J.

[15] Witjes JA, van der Heijden AG. Real world evidence of alternating cystoscopy/cytology with bladder EpiCheck in NMIBC surveillance. EAU Annual Meeting; 2021. https://www.youtube.com/watch?v=ehAvxtM0WrY.

[16] The National Comprehensive Cancer Network. NCCN clinical practice guidelines in oncology: bladder cancer version 6. 2021. https://www.nccn.org/professionals/physician_gls/pdf/bladder.pdf. 10.6004/jnccn.2024.0040 39151455

[17] Sylvester R.J., van der Meijden A.P.M., Oosterlinck W. (2006). Predicting recurrence and progression in individual patients with stage Ta T1 bladder cancer using EORTC risk tables: a combined analysis of 2596 patients from seven EORTC trials. Eur Urol.

[18] Tezak Z. Bladder EpiCheck Kit Section 510(k) premarket notification. US Food and Drug Administration; 2023. https://www.accessdata.fda.gov/cdrh_docs/pdf20/K203245.pdf.

[19] Holmang S., Johansson S.L. (2002). Stage Ta-T1 bladder cancer: the relationship between findings at first followup cystoscopy and subsequent recurrence and progression. J Urol.

[20] Blazeby J.M., Hall E., Aaronson N.K. (2014). Validation and reliability testing of the EORTC QLQ-NMIBC24 questionnaire module to assess patient-reported outcomes in non-muscle-invasive bladder cancer. Eur Urol.

[21] PROMIS. Emotional Distress-Anxiety - Short Form 4a. 2019. https://www.healthmeasures.net/index.php?option=com_instruments&view=measure&id=144&Itemid=992.

[22] Weiner B.J., Lewis C.C., Stanick C. (2017). Psychometric assessment of three newly developed implementation outcome measures. Implement Sci.

[23] Lamm D., Persad R., Brausi M. (2014). Defining progression in nonmuscle invasive bladder cancer: it is time for a new, standard definition. J Urol.

[24] Wilson C., Rooshenas L., Paramasivan S. (2018). Development of a framework to improve the process of recruitment to randomised controlled trials (RCTs): the SEAR (Screened, Eligible, Approached, Randomised) framework. Trials.

[25] Harris P.A., Taylor R., Minor B.L. (2019). The REDCap consortium: building an international community of software platform partners. J Biomed Inform.

[26] Ripping T.M., Westhoff E., Aaronson N.K. (2021). Validation and reliability of the Dutch version of the EORTC QLQ-NMIBC24 questionnaire module for patients with non-muscle-invasive bladder cancer. J Patient Rep Outcomes.

[27] Laukhtina E., Shim S.R., Mori K. (2022). Corrigendum to “Diagnostic accuracy of novel urinary biomarker tests in non-muscle-invasive bladder cancer: a systematic review and network meta-analysis” [Eur Urol Oncol 2021;4:927–42]. Eur Urol Oncol.

[28] Lotan Y., Gakis G., Manfredi M. (2021). Alternating cystoscopy with bladder EpiCheck in the surveillance of low-grade intermediate-risk NMIBC: a cost comparison model. Bladder Cancer.

[29] Schroeck F.R., St. Ivany A., Lowrance W.T., Makarov D.V., Goodney P.P., Zubkoff L. (2020). Patient perspectives on the implementation of risk-aligned bladder cancer surveillance: systematic evaluation using the tailored implementation for chronic diseases framework. JCO Oncol Pract.

[30] Holm S. (1979). A simple sequentially rejective multiple test procedure. Scand J Stat.

[31] Fine J.P., Gray R.J. (1999). A proportional hazards model for the subdistribution of a competing risk. J Am Stat Assoc.

[32] van der Aa M.N.M., Steyerberg E.W., Bangma C., van Rhijn B.W.G., Zwarthoff E.C., van der Kwast T.H. (2010). Cystoscopy revisited as the gold standard for detecting bladder cancer recurrence: diagnostic review bias in the randomized, prospective CEFUB trial. J Urol.

[33] Bluethmann S.M., Mariotto A.B., Rowland J.H. (2016). Anticipating the “silver tsunami”: prevalence trajectories and comorbidity burden among older cancer survivors in the United States. Cancer Epidemiol Biomarkers Prev.

[34] Howlader N, Noone AM, Krapcho M, et al. SEER cancer statistics review, 1975-2016. Bethesda, MD: National Cancer Institute; 2019. http://seer.cancer.gov/csr/1975_2016/.

[35] Wallace E., Higuchi R., Satya M. (2018). Development of a 90-minute integrated noninvasive urinary assay for bladder cancer detection. J Urol.

[36] Lotan Y, Roehrborn CG. Sensitivity and specificity of commonly available bladder tumor markers versus cytology: results of a comprehensive literature review and meta-analyses. Urology 2003;61:109-18; discussion 118. 10.1016/s0090-4295(02)02136-2 12559279

[37] Yafi F.A., Brimo F., Steinberg J., Aprikian A.G., Tanguay S., Kassouf W. (2015). Prospective analysis of sensitivity and specificity of urinary cytology and other urinary biomarkers for bladder cancer. Urol Oncol.

[38] Benderska-Söder N., Hovanec J., Pesch B. (2020). Toward noninvasive follow-up of low-risk bladder cancer—rationale and concept of the UroFollow trial. Urol Oncol.

