# Supplementary material for: Clinical Trial Protocol for “Replace Cysto”: Replacing Invasive Cystoscopy with Urine Testing for Non–muscle-invasive Bladder Cancer Surveillance—A Multicenter, Randomized, Phase 2 Healthcare Delivery Trial Comparing Quality of Life During Cancer Surveillance with Xpert Bladder Cancer Monitor or Bladder EpiCheck Urine Testing Versus Frequent Cystoscopy
Source: Eur Urol Open Sci. 2024 Mar 21;63:19–30. doi: 10.1016/j.euros.2024.02.018 (PMC10981003; doi:10.1016/j.euros.2024.02.018)
Supplement: Supplementary data 1 [file mmc1.pdf]

Clinical Trial Protocol for "Replace Cysto": Replacing invasive cystoscopy with urine testing for non-muscle invasive bladder cancer surveillance – a multi-center, randomized, phase 2 healthcare delivery trial comparing quality of life during cancer surveillance with Xpert® Bladder Cancer Monitor or Bladder EpiCheck® urine testing versus frequent cystoscopy

Florian R. Schroeck, MD, MS,[1, 2, 3, 4] Robert Grubb, MD,[5] Todd A. MacKenzie, PhD,[4,6] A. Aziz Ould Ismail, MD, MS,[1] Laura Jensen, MPH,[1] Gregory J. Tsongalis, PhD,[7] Yair Lotan, MD[8]

From the White River Junction VA Medical Center, White River Junction, VT[1]; Section of Urology[2] and Norris Cotton Cancer Center[3], Dartmouth Hitchcock Medical Center, Lebanon, NH; The Dartmouth Institute for Health Policy and Clinical Practice, Geisel School of Medicine at Dartmouth College, Lebanon, NH[4]; Department of Urology, Medical University of South Carolina, Charleston, SC[5] Department of Biomedical Data Science, Dartmouth College, Lebanon, NH,[6] Department of Pathology and Laboratory Medicine, Dartmouth Hitchcock Medical Center, Lebanon, NH,[7] and Department of Urology, University of Texas Southwestern, Dallas, TX[8]

Supplementary Material:

Figure S1: Word cloud with terms patients associate with cystoscopy. This was produced based on live online feedback from patients during a break-out session of the Bladder Cancer Advocacy Network's "Summit" in October 2021.

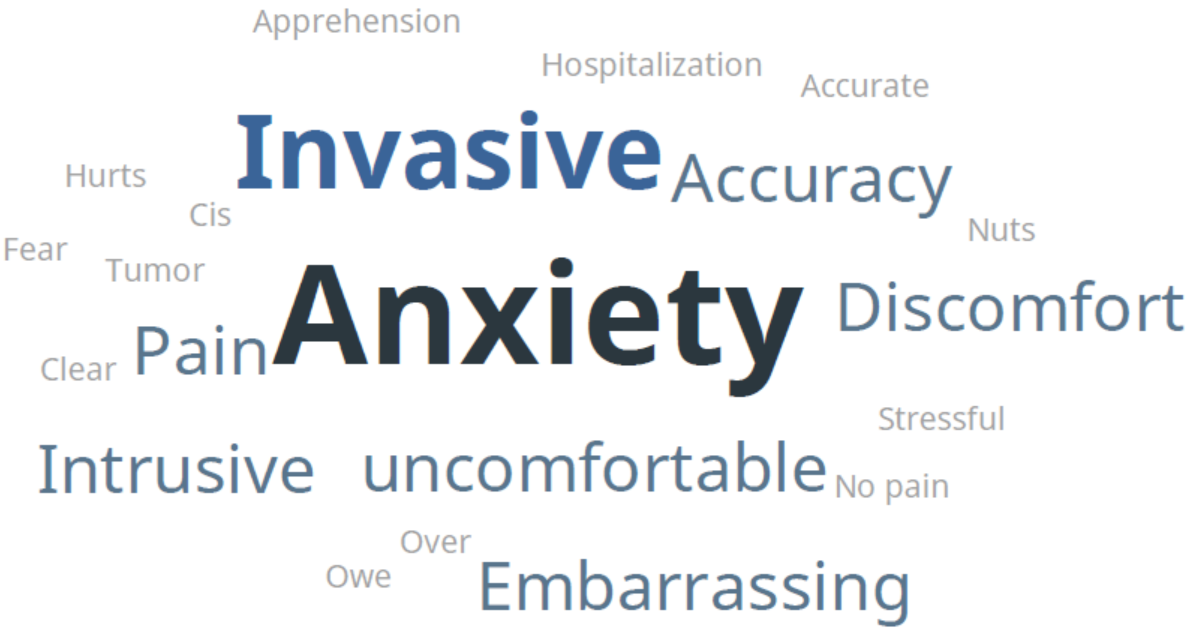

Table S1. Data supporting urologists' and patients' willingness to replace cystoscopy with urine testing.

Investigator	Setting	Main finding
Sayyid[15]	North America	75% of 51 urologists were comfortable with replacing some cystoscopy procedures with urinary testing.
Witjes[16]	Netherlands	91% of 67 patients adhered to surveillance with urine testing instead of cystoscopy when urine testing was alternated with cystoscopy (reported in abstract form).
Schroeck	Bladder Cancer Advocacy Summit	Two thirds of 22 patient/caregiver participants indicated their willingness to participate in the proposed study (unpublished preliminary data).
Schroeck	White River Junction VA	25 of 30 patients (83%) surveyed in PI Schroeck's clinic indicated they would consider participating in the proposed study (unpublished preliminary data).

Table S2. Schedule of assessments. CRF # = Case Report Form Number; S = Screening; R = Randomization; Mo = Month; Term = Termination; prn = as needed.
<u>Note:</u> *Time zero is defined as the date of the most recent bladder tumor resection. Thus, surveillance visits are labelled by months since that resection; screening and randomization will occur at the 3-month surveillance visit.*

CRF # – Study Procedures	S 3 Mo	R 3 Mo	Surveillance Visits				Term	prn
			6 Mo	12 Mo	18 Mo	24 Mo		
01 – Pre-Screening Log	X							
02 – Screening	X							
03 – Consent & Randomization		X						
04 – Surveillance Discomfort		X	X	X	X	X		
05 – Baseline, Demographics, History		X						
06 – Quality of Life & Complications		X	X	X	X	X		
07 – Surveillance Report, Procedures / Treatments since last visit			X	X	X	X		
08 – For-cause cystoscopy								X
09 – Bladder Cancer Recurrence / Progression			X	X	X	X	X	
10 – Pathology Report								X
11 – Termination							X	X
21 – AE/SAE								X
22 – AE/SAE Follow-up								X
23 – Protocol Deviation								X

RESEARCH SUBJECT CONSENT FORM

**TITLE:**	Replacing Invasive Cystoscopy with Urine Testing for Non-muscle Invasive Bladder Cancer Surveillance (Replace Cysto)
**PROTOCOL NO.:**	R37 CA275916<br>WCG IRB Protocol #20231756
**SPONSOR:**	National Cancer Institute
**INVESTIGATOR:**	Florian R. Schroeck, MD, MS<br>215 North Main Street<br>White River Junction, Vermont 05009<br>United States
**STUDY-RELATED PHONE NUMBER(S):**	802-295-9363 ext. 5368<br>802-295-9363 (24 hours)

Taking part in this research is voluntary. You may decide not to participate, or you may leave the study at any time. Your decision will not result in any penalty or loss of benefits to which you are otherwise entitled.

If you have any questions, concerns, or complaints or think this research has hurt you, talk to the research team at the phone number(s) listed in this document.

RESEARCH CONSENT SUMMARY

White River Junction Veterans Healthcare System

You are being asked for your consent to take part in a research study. This document provides a concise summary of this research. It describes the key information most people need to decide whether to take part in this research. Later sections of this document will provide all details.

What should I know about this research?

- Someone will explain this research to you.
- Taking part in this research is voluntary. Whether you take part is up to you.
- If you don’t take part, it won’t be held against you.
- You can take part now and later drop out, and it won’t be held against you.
- If you don’t understand, ask questions.
- Ask all the questions you want before you decide.

How long will I be in this research?

We expect that your taking part in this research will last 24 months.

Why is this research being done?

The purpose of this research is to determine whether bladder cancer monitoring can be improved by replacing some cystoscopy procedures with investigational urine testing.

What happens to me if I agree to take part in this research?

If you decide to take part in this research study, the general procedures include:

- You will be assigned to bladder cancer monitoring with frequent cystoscopy procedures or to a monitoring approach in which we replace every other cystoscopy procedure with a urine test.
- Regardless of which approach you are assigned to, you will have regular bladder cancer monitoring visits, similar to what you would have if you were not participating in the study.
- We will ask you about your medical history at study enrollment.
- We will ask you about discomfort experienced after each bladder cancer monitoring visit.
- We will call you a few days after completion of each bladder cancer monitoring visit. During this phone call, we ask you to complete a phone survey that will last approximately 20 to 30 minutes.
- To offset the burden of completing the surveys, you will receive $20 per completed survey as a small token of appreciation for the time, effort, and inconvenience.

What are my responsibilities if I take part in this research?

If you take part in this research, you will be responsible to:

- Keep your study appointments.
- Complete the phone surveys.
- Tell the study doctor about:
    - all medications or supplements you are taking
    - any doctors' visits or hospital stays outside of this study
    - if you have been or are currently in another research study

Could being in this research hurt me?

The most important risks or discomforts that may occur from taking part in this research include a potential delay in finding cancer that comes back in the bladder by a maximum of 6 months. This could happen with urine testing or cystoscopy, as no test is perfect. We have chosen urine tests that have been tested in thousands of patients. Based on this testing, it is very rare that cancer is missed with these tests but there is no guarantee. At first glance, it may seem like cystoscopy is a perfect test to find changes within the bladder. However, we know from various studies that tumors can be missed with cystoscopy. In addition, you are eligible for this study because you have a low-grade bladder cancer, which is a type of cancer, where a small delay would be unlikely to cause any serious harm.

You might also experience discomfort after cystoscopy procedures done for bladder cancer monitoring. However, these procedures will be no different than the procedures we would do if you were not participating in the study.

Will being in this research benefit me?

We cannot guarantee any benefits in the study. The most important benefits that may occur from taking part in this research include the possibility of undergoing bladder cancer monitoring with urine tests. These urine tests are not offered outside of the research setting. If you are assigned to urine testing, you will need fewer cystoscopy procedures and might have less discomfort due to needing fewer procedures.

Possible benefits to others in the future include that the results from this study – if positive – may provide more evidence on the potential benefits of bladder cancer monitoring with urine testing.

The advantages and disadvantages of the monitoring approaches are summarized in Table 1.

***Table 1**. Advantages and disadvantages of the monitoring approaches*

	**Urine Testing Arms (Xpert or EpiCheck urine tests)**	**Frequent Cystoscopy Arm**
Advantages	• Fewer invasive cystoscopy procedures. This may lead to less discomfort & anxiety.	• More frequent look directly into the bladder.<br>• This may give you peace of mind.
Disadvantages	• Urine tests may miss cancer in approximately 30 out of 100 patients who have a recurrent tumor.<br>• Because tumor recurrence is rare, only 1 to 8 out of 100 participants are expected to have a tumor missed by urine testing.	• Cystoscopy may miss cancer in approximately 30 out of 100 patients who have a recurrent tumor.<br>• Because tumor recurrence is rare, only 1 to 8 out of 100 participants are expected to have a tumor missed by cystoscopy.

What other choices do I have besides taking part in this research?

Instead of being in this research, you may decide to have bladder cancer monitoring outside of the study. This will likely entail monitoring with regular cystoscopy procedures and without urine testing.

DETAILED RESEARCH CONSENT

You are being invited to take part in a research study. A person who takes part in a research study is called a research participant.

What should I know about this research?

- Someone will explain this research to you.
- This form sums up that explanation.
- Taking part in this research is voluntary. Whether you take part is up to you.
- You can choose not to take part. There will be no penalty or loss of benefits to which you are otherwise entitled.
- You can agree to take part and later change your mind. There will be no penalty or loss of benefits to which you are otherwise entitled.
- If you don’t understand, ask questions.
- Ask all the questions you want before you decide.

Why is this research being done?

The purpose of this research is to determine whether bladder cancer monitoring can be improved by replacing some cystoscopy procedures with investigational urine testing. Specifically, we are examining whether there are any differences in urinary symptoms, discomfort, number of invasive procedures, anxiety, complications, cancer recurrence or cancer progression when some cystoscopy procedures are replaced with urine testing.

About 240 participants will take part in this research.

How long will I be in this research?

We expect that your taking part in this research will last approximately 24 months.

What happens to me if I agree to take part in this research?

The only way we can compare monitoring with Frequent Cystoscopy and monitoring with urine testing directly and find out more about patients' experiences and outcomes is to do a study called a "randomized trial." This study will have three groups, also called "arms": (1) Frequent Cystoscopy Arm, (2) Xpert Urine Test Arm, and (3) EpiCheck Urine Test Arm. We need to divide patients into three groups that are as identical as possible except that one group is assigned to the Frequent Cystoscopy Arm, one to the Xpert Urine Test Arm, and one to the EpiCheck Urine Test Arm. The groups need to have identical numbers of younger/older and taller/shorter people, and so on. If the treatment groups are the same as each other, this allows a fair comparison. If you or your doctor choose a monitoring approach, the groups will not end up the same and then the comparison would not be fair.

If you take part in this study, you could be assigned to any of the three arms. The process of randomization means you have an equal chance of being assigned to each arm, or a two out of three chance to be assigned to one of the two urine testing arms. If you think you have a strong preference for Frequent Cystoscopy or urine testing, it is important that you tell the study doctor or study coordinator. You are being offered the study because your doctor believes that both approaches are appropriate options for you. You will be able to ask all the questions you have and discuss randomization in more detail with the study team or your doctor.

What are the monitoring approaches offered in the study?

There are three monitoring approaches in this study:
(1) Frequent Cystoscopy Arm
(2) Xpert Urine Test Arm
(3) EpiCheck Urine Test Arm

More details are described below and in Figure 1.

<u>Frequent Cystoscopy</u>
The aim of Frequent Cystoscopy is to detect any cancer that might have come back within the bladder by frequently inspecting the bladder. You will have a cystoscopy procedure at specified time points for two years.

If any changes are detected during these check-up visits, your doctor and their clinical team will discuss further evaluation, for example with biopsy procedures or imaging studies. If cancer is found in your bladder at any time, you will undergo standard treatment and you will leave the study after that treatment.

Cystoscopy is a direct inspection of the bladder. At first glance, it may seem like cystoscopy is a perfect test to find changes within the bladder. However, we know from various studies that tumors can be missed with cystoscopy. We don't know exactly how often tumors are missed with cystoscopy, but from the available data this may happen in approximately 30 out of 100 patients who have a recurrent tumor.

However, because most patients will not have a recurrent tumor, only 1 to 8 out of 100 patients in this study are expected to have a tumor missed by cystoscopy but there is no guarantee. To assure participants' safety, the study will be regularly monitored by a Safety Monitoring Committee.

Xpert Urine Test
The aim of monitoring in this arm is to detect any cancer that might have come back within the bladder, while decreasing the number of invasive cystoscopy procedures. You will have a Xpert urine test and a check-up with your doctor at 6 months and 18 months. You will have a cystoscopy procedure at 12 months and 24 months.

If any changes are detected during these check-up visits, your doctor and their clinical team will discuss further evaluation, for example with cystoscopy procedures, biopsy, or imaging studies. If cancer is found in your bladder at any time, you will undergo standard treatment and you will leave the study after that treatment.

The Xpert Urine Test is investigational, which means that it is not approved by the Food and Drug Administration (FDA). Thus, this urine test is currently not available outside of the study setting. When you have completed the study, you may not be able to get this urine test as part of your routine care.

Based on the available data, we estimate that a tumor may be missed with the Xpert urine test in approximately 30 out of 100 patients who have a recurrent tumor. However, because most patients will not have a recurrent tumor, only 1 to 8 out of 100 patients in this study are expected to have a tumor missed by urine testing but there is no guarantee. To assure participants' safety, the study will be regularly monitored by a Safety Monitoring Committee.

EpiCheck Urine Test
The aim of monitoring in this arm is to detect any cancer that might have come back within the bladder, while decreasing the number of invasive cystoscopy procedures. You will have an EpiCheck urine test and a check-up with your doctor at 6 months and 18 months. You will have a cystoscopy procedure at 12 months and 24 months.

If any changes are detected during these check-up visits, your doctor and their clinical team will discuss further evaluation, for example with cystoscopy procedures, biopsy, or imaging studies.

If cancer is found in your bladder at any time, you will undergo standard treatment and you will leave the study after that treatment.

The EpiCheck Urine Test is a laboratory-developed test for early-stage non-muscle invasive bladder cancer monitoring. The test was developed and its performance determined exclusively by Nucleix Inc. The EpiCheck Urine Test is investigational, which means that it is not approved by the Food and Drug Administration (FDA). When you have completed the study, you may not be able to get this urine test as part of your routine care.

Based on the available data, we estimate that a tumor may be missed with the EpiCheck urine test in approximately 30 out of 100 patients who have a recurrent tumor. However, because most patients will not have a recurrent tumor, only 1 to 8 out of 100 patients in this study are expected to have a tumor missed by urine testing but there is no guarantee. To assure participants' safety, the study will be regularly monitored by a Safety Monitoring Committee.

Figure 1 on the next page summarizes the monitoring and the monitoring visits during this study. In addition:

- We will collect medical information from your medical record.
- We will ask you about your medical history at study enrollment.
- We will ask you about discomfort experienced after each bladder cancer monitoring visit.
- We will call you a few days after completion of each bladder cancer monitoring visit to complete a phone survey that will last approximately 20 to 30 minutes.

To offset the burden of completing the surveys, you will receive $20 per completed survey as a small token of appreciation for the time, effort, and inconvenience.

Figure 1: The 3 monitoring approaches used in this study and subsequent follow-up visits with data collection.

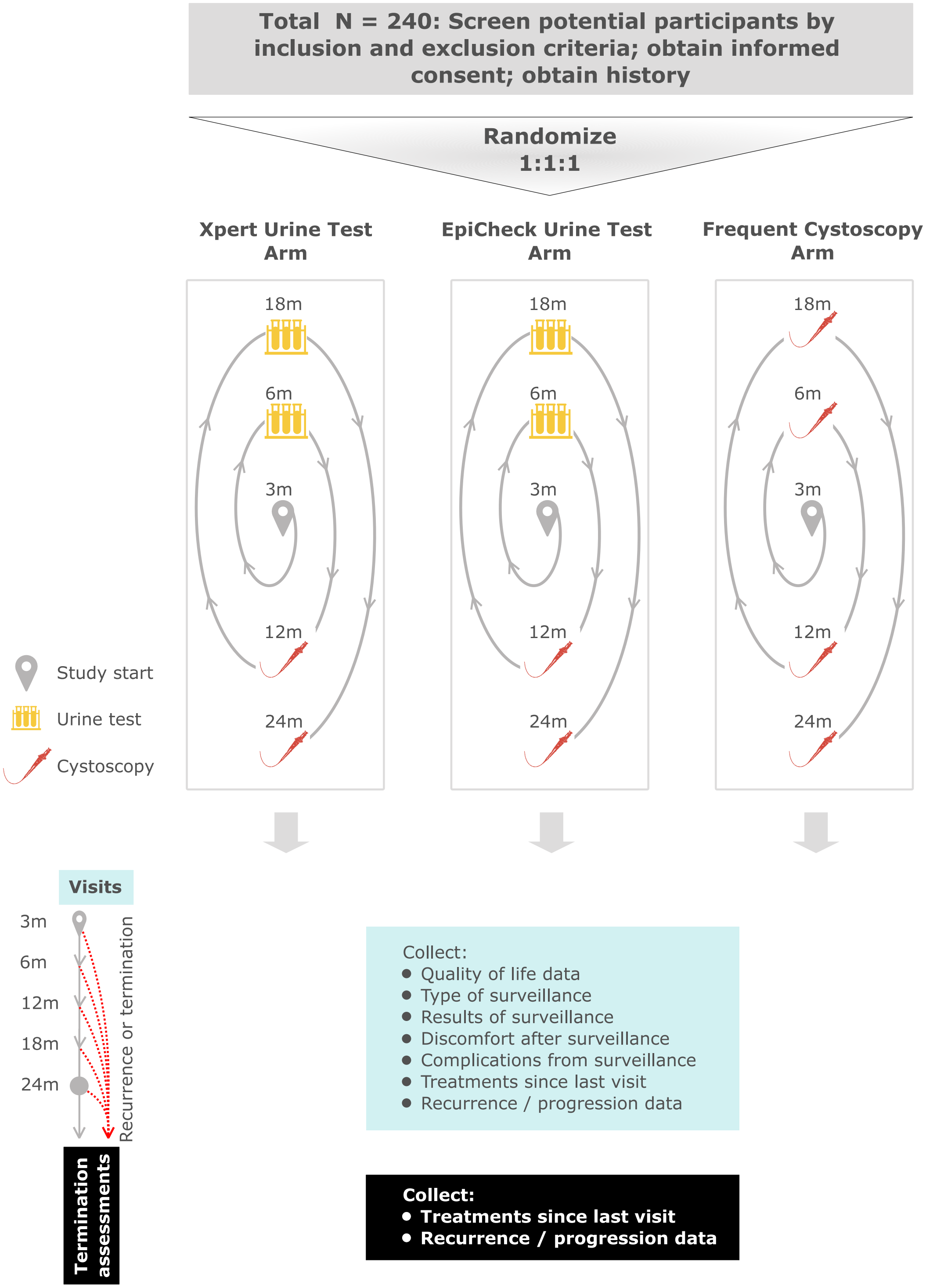

What are my responsibilities if I take part in this research?

If you take part in this research, you will be responsible to:

- Keep your study appointments.
- Complete the phone surveys.
- Tell the study doctor about:

- all medications or supplements you are taking
- any doctors' visits or hospital stays outside of this study
- if you have been or are currently in another research study

Could being in this research hurt me?

Risks or discomforts that you may expect from taking part in this research include:

- A potential delay in finding cancer that comes back in the bladder by a maximum of 6 months. This could happen with urine testing and with cystoscopy as no test is perfect. We have chosen urine tests that have been tested in thousands of patients. Based on this testing, it is very rare that cancer is missed with these tests but there is no guarantee. At first glance, it may seem like cystoscopy is a perfect test to find changes within the bladder. However, we know from various studies that tumors can be missed with cystoscopy. In addition, you are eligible for this study because you have a low-grade bladder cancer, which is a type of cancer, where a small delay would be unlikely to cause any serious harm.
- Discomfort after cystoscopy procedures done for bladder cancer monitoring. The risks of cystoscopy procedures include infection, bleeding, and urinary retention due to irritation and swelling from the procedure. However, these procedures will be no different than the procedures we would do if you were not participating in the study.
- Urinary symptoms after cystoscopy procedures done for bladder cancer monitoring. However, these procedures will be no different than the procedures we would do if you were not participating in the study.
- Questions of the surveys may include sensitive or private topics that you may not normally discuss. Answering such questions could potentially cause distress. Please tell the study doctor or study staff if you feel uncomfortable or upset while answering survey questions. You have the right to refuse to answer any questions. While your direct responses to the survey will not be shared with the study team (study doctor or study staff), if you are experiencing substantial worry or anxiety, we will alert your study team. We create research records of care for this study. Many steps have been taken to safeguard these records. Efforts will be made to limit your personal information to people who have a need to review this information. However, in spite of these steps and efforts, a small privacy risk remains, for example private information could be accidentally disclosed. As a National Institutes of Health-funded study, this study is issued a Certificate of Confidentiality. This means that identifiable research information is protected from forced disclosure. It allows the investigator and others who have access to research records to refuse to disclose identifying information on research participation in any civil, criminal, administrative, legislative, or other proceeding, whether at the federal, state, or local level. However, you may choose to voluntarily disclose the protected information and this certificate does not prohibit such voluntary disclosure. Furthermore, the parties listed in the Confidentiality / Authorization section of this consent form may review our records under limited circumstances and this certificate does not prohibit such disclosure.

In addition to these risks, taking part in this research may harm you in unknown ways.

What if I am injured because of taking part in this research?

If you have an injury or illness as a result of being in this study, the VA will provide emergency treatment and medical care at no cost to you. No additional payments by the VA are planned. By signing this form, you do not lose any of your legal rights or release the VA Healthcare System from its duty to provide proper medical care.

If you believe you have injury, illness, or a bad reaction related to the study activities, please let the researcher know as soon as possible. You can call the researcher at (802) 295-9363 ext. 5368 during the day or the on-call VA doctor at (802) 295-9363 after business hours. If you receive emergency medical care in a private hospital because you are unable to come to the VA Medical Center, please have a family member or friend let the study staff know. The VA Medical Center can then work with the private hospital to support your medical care.

More information about medical care and payments for medical services in the event of a study related injury or illness is available from the VA Healthcare System's Business Office at (802) 295-9363 extension 4151. You may also call the VA Healthcare System's Patient Advocate at (802) 295-9363 extension 6293.

Will it cost me money to take part in this research?

You will not be charged for the Xpert or EpiCheck urine tests if you are assigned to a urine testing arm. However, if you usually pay co-payments for VA care and medications, you will still pay these co-payments for your VA bladder cancer care (*e.g.*, cystoscopy procedures and bladder cancer treatments).

Will being in this research benefit me?

We cannot promise any benefits to you or others from your taking part in this research. However, possible benefits to you include the possibility of undergoing bladder cancer monitoring with urine tests. These urine tests are not offered outside of the research setting. If you are assigned to urine testing, you will need fewer cystoscopy procedures and might have less discomfort due to needing fewer procedures.

Possible benefits to others in the future include that the results from this study – if positive – may provide more evidence on the potential benefits of bladder cancer monitoring with urine testing.

What other choices do I have besides taking part in this research?

If you choose not to take part, your healthcare or benefits will not be affected. If you decide to not take part in this study, you will likely undergo Frequent Cystoscopy. However, you can also discuss the following options with your treating doctor:

- You may choose to undergo no monitoring of your bladder cancer.
- You may choose to take part in a different study if one is available.

What happens to the information collected for this research?

Your private information and your medical record will be shared with individuals and organizations that conduct or watch over this research, including:

- The research sponsor,
- People who work with the research sponsor,

- People who work at the coordinating site, which is the Veterans Education & Research Association of Northern New England (VERANNE) & the White River Junction VA Healthcare System in Vermont,
- The laboratories running the urine tests will receive your identifying information so they can run the tests and report back the results,
- People who work at Dartmouth College, who coordinate the data collection and data analyses for this study,
- The institution where the research is being done,
- Government agencies, such as the Food and Drug Administration, the Office of Human Research Protections, the VA Office of Research Oversight, or the Government Accountability Office,
- WCG IRB, the Institutional Review Board (IRB) that reviewed this research.

Your data will also be shared with central research team members at the Veterans Education & Research Association of Northern New England (VERANNE) and the White River Junction VA Healthcare System, Vermont. These central research team members are responsible for running the overall study. Most of this data will be coded and identified only by a research identification number. However, some identifiable information may be shared as well, for example to allow the central research team to process your compensation for completing the phone surveys.

Coded data identified only by a research identification number will also be shared with Dartmouth College. Dartmouth College coordinates the data collection and data analyses for this study.

The laboratories running the urine tests will receive your identifying information so they can run the tests and report back the results. The Xpert Bladder Cancer Monitor urine test will be run at the Dartmouth Hitchcock Pathology Laboratory in Lebanon, New Hampshire, and the EpiCheck urine test will be run at the Nucleix Inc. Laboratory within the United States. Both labs are certified to perform high quality and safe patient testing (so-called CLIA certification).

We may publish the results of this research. However, we will keep your name and other identifying information confidential.

We protect your information from disclosure to others to the extent required by law. We cannot promise complete secrecy.

A description of this clinical trial will be available on http://www.ClinicalTrials.gov, as required by U.S. Law. This Web site will not include information that can identify you. At most, the Web site will include a summary of the results. You can search this Web site at any time.

Data collected in this research might be deidentified and used for future research or distributed to another investigator for future research without your consent. Per National Cancer Institute (NCI) requirements, the de-identified data will be transmitted to an NCI-approved data service.

Urine samples may be used for commercial profit, and you will not share in this commercial profit. The clinically relevant research results may not be disclosed with you. The urine samples will not be used for whole genome sequencing.

What information may be used and given to others?

The study doctor will get your personal and medical information. For example:

- Past and present medical records,
- Research records,
- Records about phone calls made as part of this research,
- Records about your study visits,
- Results from urine testing if you are in one of the urine testing arms.

Who may use and give out information about you?

The study doctor and the study staff.

Why will this information be used and/or given to others?

- to do the research,
- to study the results, and
- to make sure that the research was done right.

If the results of this study are made public, information that identifies you will not be used.

What if I decide not to give permission to use and give out my health information?

Then you will not be able to be in this research study.

May I review or copy my information?

Yes, but only after the research is over.

Is my health information protected after it has been given to others?

All others that receive your information have policies in place to protect your data. However, there remains a small risk that your information will be given to others without your permission.

Treatment, payment or enrollment/eligibility for benefits cannot be conditioned on you signing this authorization. This authorization will expire at the end of the research study unless revoked prior to that time.

May I withdraw or revoke (cancel) my permission?

Yes, but this permission will not stop automatically. When you withdraw your permission, no new health information identifying you will be gathered after that date. Information that has already been gathered may still be used and given to others.

You may withdraw or take away your permission to use and disclose your health information at any time. If you withdraw your permission, you will not be able to stay in this study.

Withdrawing your permission will not affect your VA healthcare, including your doctor's ability to see your records as part of your normal care and will not affect your right to have access to the research records after the study is completed.

If you decide to withdraw your permission, contact Florian Schroeck, MD, MS, the study's principal investigator.

Can I be removed from this research without my approval?

The person in charge of this research can remove you from this research without your approval. Possible reasons for removal include:

- It is in your best interest
- The research is canceled by the FDA or the sponsor
- You are unable to keep your scheduled appointments
- You have a bladder cancer recurrence requiring biopsy or resection
- You develop a condition such as pregnancy that precludes further study participation

We will tell you about any new information that may affect your health, welfare, or choice to stay in this research.

What happens if I agree to be in this research, but I change my mind later?

If you decide to leave this research, contact the research team so that the investigator can:

- Discuss with you what kind of care you would receive outside of the study
- Determine the reason why you decided to leave this research

Will I be paid for taking part in this research?

For taking part in this research, you may be paid up to a total of $100. Your compensation will be broken down as follows:

- You will receive $20 after each phone survey you completed
- There are up to five surveys over the course of 2 years. Thus, you may be paid up to $100
- You will receive payment from the central study coordinating team at the Veterans Education & Research Association of Northern New England (VERANNE) and the White River Junction VA Healthcare System, Vermont

Who can answer my questions about this research?

If you have questions, concerns, or complaints, or think this research has hurt you or made you sick, talk to the research team at the phone number listed above on the first page.

This research is being overseen by WCG IRB. An IRB is a group of people who perform independent review of research studies. You may talk to them at 855-818-2289 or email researchquestions@wcgirb.com if:

- You have questions, concerns, or complaints that are not being answered by the research team.
- You are not getting answers from the research team.

- You cannot reach the research team.
- You want to talk to someone else about the research.
- You have questions about your rights as a research subject.

Statement of Consent:

Your signature documents your consent to take part in this research.

Signature of adult participant capable of consent	Date
Signature of person obtaining consent	Date
